# Migrant workers’ health intermediarization: Institutional intermediaries in the social ecologies of Taiwan and Japan

**DOI:** 10.1016/j.jmh.2026.100463

**Published:** 2026-11-05

**Authors:** Yen-Ru Chen, Po-Han Lee

**Affiliations:** a Institute of Health Policy and Management, College of Public Health, National Taiwan University, Taipei, Taiwan; b Global Health Program, College of Public Health, National Taiwan University, Taipei, Taiwan

**Keywords:** Health intermediarization, Migrant health, Social ecological model (SEM), Intermediaries, Labor migration

## Abstract

**Background:**

Research on labor migration has documented the roles of intermediaries in recruitment and settlement, yet their influence on migrant workers’ health—particularly healthcare access and navigation—remains underexplored. This study examines the process of *health intermediarization* among migrant workers in Taiwan and Japan.

**Methods:**

Using the Social Ecological Model (SEM) as an analytical framework, this qualitative study adopts a parallel case study design to examine how intermediaries are institutionally positioned within migrant health navigation. Data were drawn primarily from individual interviews and group discussions with scholars and practitioners knowledgeable about Taiwan’s private intermediary system and Japan’s Technical Intern Training Program (TITP) and Specified Skilled Worker (SSW) system, supplemented by field-based interactions, expert consultations, and contextual policy materials.

**Results:**

Across both contexts, intermediaries—while not healthcare providers—shape migrant health navigation by controlling information flows, managing administrative procedures, and mediating access to resources. In Taiwan, private intermediaries coordinate documentation, communication, and everyday arrangements that condition care-seeking. In Japan, supervising and support organizations provide procedural guidance and adaptation support within formalized migration programs. These arrangements shape when, how, and whether migrant workers are able to access healthcare services.

**Conclusions:**

This study conceptualizes these dynamics as *health intermediarization*: an institutional process through which intermediary arrangements mediate health-related decisions and access pathways. By foregrounding institutional embeddedness within SEM-informed analysis, the findings highlight the under-recognized role of intermediary arrangements in shaping migrant health navigation and healthcare access.

## Highlights

• Migrant healthcare access is co-shaped by intermediary-mediated navigation.
• Intermediaries structure health decisions by informational and procedural access.
• In Taiwan, private brokers embed health navigation within coordination practices.
• In Japan, supervising and support organizations mediate health navigation through formal migration programs.
• Intermediaries are a meso-level determinant of migrant health in social ecology.

## 1 Introduction

International labor migration occurs through diverse governance arrangements that shape how recruitment, placement, and ongoing coordination are organized. In many Asian migration systems, these arrangements take an intermediary form, involving private agencies, employers, and regulatory actors working across borders (Andrees et al., 2015; International Labour Organization, 2022; OECD et al., 2024). In such contexts, intermediaries shape recruitment conditions, workplace coordination, and the oversight or support workers receive (Gordon, 2015; Piper, 2017).

Migration scholarship has conceptualized intermediaries as assemblers linking heterogeneous infrastructures (Lindquist et al., 2012), as actors embedded within broader migration infrastructures (Xiang and Lindquist, 2014), and as coordinators of documentation and communication across regulatory spaces (Hernández-León, 2021; McKeown, 2012). Existing studies have examined recruitment experiences, indebtedness, matching processes, and access to assistance (Lan and Chien, 2022; Liang, 2019). However, intermediary involvement is often treated as a set of discrete functions rather than as a continuous institutional presence shaping migrants’ everyday environments.

Research on migrant health has shown that decisions to seek or forgo care are embedded in employment insecurity, insurance arrangements, and institutional constraints. In these contexts, intermediaries often feature in the routine organizational landscape through their roles regarding information provision and procedural coordination, yet their influence on health navigation has rarely been analytically foregrounded (Antonio and Lee, 2024; Miller et al., 2022).

This issue is particularly salient in Asia, where dense brokerage networks underpin temporary migration flows (Kneebone, 2010; Martin, 1991). The present study focuses on documented migrant workers entering through formally regulated, intermediary-intensive labor-migration pathways in Taiwan and Japan. These arrangements primarily concern temporary migrant labor and differ from those governing undocumented migration or more highly skilled forms of labor mobility. In Taiwan, private intermediaries link origin brokers, employers, and state agencies, coordinating documentation, communication, and settlement tasks (Lan and Chien, 2022; Lin, 2014). In Japan, the Technical Intern Training Program (TITP) and the Specified Skilled Worker (SSW) system involve intermediary arrangements through supervising and support organizations responsible for procedural coordination, interpretation, and settlement-related assistance (Inaba, 2023; Lan, 2022; Ling and Hsiao, 2019).

Against this background, this study addresses two gaps. First, despite extensive research on intermediaries in recruitment and labor governance, little attention has been paid to how intermediary involvement shapes migrants’ health-seeking and healthcare navigation processes. Second, applications of the Social Ecological Model (SEM) to migrant health have rarely examined intermediaries within the organizational environments through which care navigation unfolds. Drawing on qualitative data from Taiwan and Japan, contextualized by relevant policy and administrative materials, this study examines how intermediary arrangements are institutionally embedded within migrant health navigation and conceptualizes these dynamics as a process of *health intermediarization*.

In this study, health intermediarization refers to the institutional process through which intermediary arrangements organize and shape migrants’ practical navigation of health-related information, procedures, communication, and assistance. These arrangements may involve diverse actors and organizational forms—including individual brokers, private agencies, public or quasi-public bodies, public–private arrangements, and NGOs—that they mediate between migrants and the institutions or resources relevant to health-related needs. We use the term intermediary environment to describe the organizational configuration within which this process occurs, rather than as a synonym for health intermediarization itself.

## 2 Conceptual extension: multilevel perspectives on migrant health

Temporary labor migration exposes workers to a range of health-related risks and constraints that cannot be understood at the individual level alone. Migrant health scholarship has therefore increasingly drawn on socio-ecological perspectives to conceptualize well-being as shaped by interactions across individual, relational, institutional, and structural domains. The SEM frames health not as an outcome of personal behaviors, but as produced through layered environments conditioning risks, resources, and capabilities (Bronfenbrenner, 1994; Richard et al., 2011).

Applied to migration contexts, SEM-informed analyses demonstrate how interpersonal relations, workplace arrangements, service access, and broader sociopolitical conditions jointly influence physical and psychological well-being. For example, research on migrant trauma highlights how psychological distress is shaped by precarious labor conditions, discrimination, and limited institutional support rather than individual experience alone (Rivera, 2023). Studies of healthcare navigation among migrant women similarly show how provider communication, organizational practices, and clinical protocols intersect to shape access to care (Goodwin et al., 2022; Souleymanov et al., 2023). Other work emphasizes how transportation, employment conditions, and bureaucratic systems structure migrants’ practical ability to navigate health services (Moyce et al., 2023).

The literature review confirms the value of SEM for interpreting migrant health as embedded within multilevel environments. At the same time, meso-level influences within SEM are most often conceptualized through healthcare institutions, community resources, and settlement infrastructures. Comparatively less attention has been paid to organizational actors embedded within labor migration systems—such as brokers, placement agents, supervising bodies, and support organizations—despite their routine structuring of migrants’ daily living, working, and assistance-seeking environments.

This gap reflects not the limitation of SEM itself, but a lack of analytic integration of intermediary arrangements as meso-level organizational contexts through which migrants manage risks, constraints, and health-related decisions. Recognizing intermediary arrangements as such contexts opens analytical space to examine them not merely as facilitators of labor mobility, but as institutionally embedded environments with everyday relevance for migrant health and well-being.

## 3 Theoretical framing: intermediaries’ institutional embeddedness

Theorizing brokerage as an institutional environment rather than a transactional function shifts analytical attention from recruitment events to the organizational infrastructures through which migrants routinely interact with formal systems. Migration infrastructure scholarship shows that intermediaries coordinate documentation, communication, expectations, procedural interpretation, and affective labor, thereby structuring the routes migrants navigate through institutions (Lindquist et al., 2012; Xiang and Lindquist, 2014). Rather than acting as isolated brokers, they assemble dispersed legal, informational, and relational resources into navigable channels linking employers, state agencies, and migrants (Koster and Van Leynseele, 2018). Their embeddedness reflects the distributed nature of procedural tasks across governance actors, so no single institutional site fully encompasses these functions (Hernández-León, 2021; McKeown, 2012).

Across Asian migration regimes, this embeddedness is structural rather than incidental. Intermediaries stabilize uncertainty, facilitate information flows, and orchestrate procedural compliance within fragmented administrative arenas (Kneebone, 2010; Martin, 1991; OECD et al., 2024). Recruitment is only one domain of this work; brokerage also encompasses document management, administrative translation, dispute interpretation, and mediating expectations about rights and obligations (Andrees et al., 2015; Gordon, 2015). Seen in this way, intermediaries function as infrastructures of governance rather than solely as market actors, rendering opaque formal systems practically actionable for migrants.

This conceptual positioning highlights that intermediaries occupy meso-level institutional spaces that mediate between governance structures and lived experience. They shape what actions are feasible, intelligible, or delayed—not by providing services themselves, but by organizing the procedural conditions under which decisions are made. This study does not argue that intermediaries are health actors; instead, it argues that health navigation is institutionally routed through them. Recognizing intermediary arrangements as organizational environments does not imply a uniform or deterministic impact, but it enables analysis of how routinized procedures and distributed responsibility shape migrant agency. The analysis that follows examines how institutional embeddedness positions intermediaries as infrastructural components that mediate migrants’ interactions with bureaucratic systems, employers, and everyday needs, using the Taiwan and Japan cases to empirically illustrate these dynamics without implying system equivalence.

## 4 Mediating health access: intermediaries as institutional contexts

Intermediaries are not conventionally conceptualized as health actors, and migration scholarship has primarily examined their roles in recruitment, documentation, communication, and everyday coordination rather than in terms of health outcomes (Lindquist et al., 2012; McKeown, 2012; Xiang and Lindquist, 2014). Nevertheless, these institutional roles place intermediaries at key points of interaction where migrants interpret regulations, respond to administrative demands, and negotiate uncertainty. Although not designed as health-related functions, such activities shape the environments through which migrants determine what actions are feasible, intelligible, or deferred.

Across Asian migration regimes, intermediaries occupy these positions not because they replace state or employer institutions, but because practical governance is dispersed and procedural responsibilities do not reside in any single organizational site (International Labour Organization, 2022; Martin, 1991; OECD et al., 2024; Xiang and Lindquist, 2014). In Taiwan, private agencies routinely coordinate settlement, communication, and everyday procedures within the migration regime, functioning as institutional interlocutors rather than formal welfare actors (Lan and Chien, 2022; Liang, 2019; Lin, 2014). In Japan, studies of the Technical Intern Training Program (TITP) and the Specified Skilled Worker (SSW) system describe supervising and support organizations that provide procedural guidance, language assistance, and coordination of daily practical matters, including those related to health and safety management (Aida and Mori, 2021; Tsujimura, 2020; Lan, 2022; Ishimaru et al., 2023).

From a socio-ecological perspective, these arrangements constitute meso-level organizational settings linking migration governance with migrants’ everyday navigation of information, assistance, and services. Their relevance lies not in functioning as healthcare providers, but in shaping the practical conditions under which health-related options become available, intelligible, accessible, constrained, or deferred. Thus, examining intermediaries in this way brings into view an institutional dimension of migrant health navigation that has remained underexplored and under-described in conventional applications of the SEM, while allowing for variation in how intermediary arrangements operate across different migration systems.

## 5 Methods

This study employed a qualitative, case-oriented design to examine how intermediary arrangements shape migrant workers’ capacities to navigate health-related needs within formally regulated labor-migration pathways. Taiwan and Japan were selected as analytically parallel cases because both institutionalize intermediary involvement in labor migration, albeit through distinct governance mechanisms. In Taiwan, private brokerage agencies coordinate recruitment and provide ongoing administrative and logistical support. In Japan, comparable functions are embedded within formally regulated arrangements involving Supervising Organizations under the TITP and Registered Support Organizations under the SSW system. The two settings were examined as separate institutional contexts rather than as a comparative case pair to illustrate how different configurations position intermediaries within migrants’ everyday navigation environments.

### 5.1 Design and materials

Qualitative data were collected between March and April 2024. Because the study sought to understand the institutional organization of intermediation rather than estimate prevalence or systematically document individual migrant experiences, recruitment prioritized participants with institutional expertise or frontline knowledge of migrant labor, health, and intermediary arrangements. Initial participants were identified purposively, with subsequent recruitment also occurring through participant referrals and field-based introductions. The two primary participant groups were selected for complementary forms of institutional knowledge. Japanese scholars provided expertise on the policy and organizational architecture of the TITP and SSW systems, while Taiwanese NGO practitioners contributed frontline knowledge of how intermediary arrangements operate in employment and healthcare contexts. The study did not recruit intermediary organizations themselves; its primary aim was to examine how intermediaries are positioned within broader systems of migration and health governance rather than to document intermediaries’ self-understandings.

We conducted the Japanese individual interview and multi-participant group discussions online, while data collection in Taiwan included both face-to-face and online interviews. Interviews and group discussion sessions generally lasted approximately one hour. In Japan, data collection began with one individual interview and, following a snowballing process, subsequently expanded to four online group discussions at invited participants’ request. Public health scholars with expertise in migrant health, as well as the TITP and SSW systems, participated in these sessions. In Taiwan, we interviewed two NGO representatives individually, drawing on their firsthand experience helping migrant workers navigate employment, administrative, and health-related systems.

Following the Taiwan interviews, the NGO representatives facilitated access to migrant community settings, where field-based conversations were conducted with a community informant and a migrant worker. Taiwanese scholars specializing in migrant and occupational health were subsequently consulted, with consent, to clarify institutional arrangements and interpretations emerging from the fieldwork. The first author conducted the formal interview and group discussions with Japanese participants in English, and interviews with Taiwanese NGO practitioners and subsequent scholar consultations in Mandarin Chinese. During the field-based conversation with the migrant participant, interpretation support was provided by the accompanying NGO practitioner.

Data collection in both countries addressed migrant workers and intermediary arrangements broadly, rather than focusing on particular occupational sectors. The Taiwan materials nevertheless included sector-specific examples concerning industrial work, domestic and care work, and fisheries. Discussions in Japan centered predominantly on formally employed migrant workers in service and industrial sectors; fisheries and live-in care work were not substantively represented. Occupational differences are therefore treated as contextual variation rather than as a basis for systematic cross-sector comparison. Table 1 presents the anonymized participant codes, their roles, and their contributions to the study:

**Table 1:** Study participants, institutional relevance, and mode of contribution.

Code	Participant Type	Country	Role and Relevance	Mode of contribution
J-1	Scholar	Japan	Researcher specializing in migrant health, with expertise in migrant workers under Japan’s TITP.	Individual interview/group discussions
J-2	Scholar	Japan	Public health scholar focusing on migrant health issues in Japan, including access to healthcare and occupational conditions.	Group discussions
J-3	Scholar	Japan	Public health researcher with expertise in migrant worker health and health systems in Japan.	Group discussions
J-4	Scholar	Japan	Public health scholar studying health and welfare issues among migrant workers in Japan.	Group discussions
J-5	Scholar	Japan	Occupational health expert with research experience on Japan’s migrant labor system and migrant worker health.	Group discussions
T-1	Scholar	Taiwan	Researcher specializing in migrant worker health in Taiwan, with a focus on occupational health.	Expert consultation
T-2	Community informant (Indonesian)	Taiwan	Community-based informant engaged in supporting migrant workers’ daily needs and welfare within migrant communities in Taiwan.	Field-based conversation
T-3	NGO Representative	Taiwan	NGO practitioner providing frontline support to migrant workers across different employment sectors, including industrial and domestic work.	Formal interview
T-4	NGO Representative	Taiwan	NGO practitioner working primarily with migrant fishery workers, with experience supporting health and labor-related navigation.	Formal interview
T-5	Scholar	Taiwan	Public health scholar with expertise in occupational health and research experience on migrant health in Taiwan.	Expert consultation
T-6	Migrant Worker	Taiwan	Migrant fishery worker with experience in long-distance fishing operations.	Field-based conversation

Note: J-1 first participated in an individual interview, and J-1–J-5 participated in all four group discussions; accordingly, group-discussion findings are attributed to participants rather than to individual discussion sessions.

The anonymized designations attribute viewpoints while maintaining participant confidentiality. We also consulted official policy and administrative documents to support institutional contextualization—e.g., program rules, official guidance, and administrative information on recruitment/intermediation and the TITP/SSW arrangements. In Taiwan, government materials on migrant labor recruitment and intermediary arrangements were reviewed prior to data collection to inform the interview guide and understanding of the institutional context. In Japan, we reviewed relevant official materials on the TITP and SSW systems after the interviews to clarify the institutional arrangements participants described and support interpretation of the qualitative data. We did not independently code these materials or treat them as a separate documentary dataset; instead, we used them to contextualize institutional arrangements, clarify participant accounts, and check interpretations of program structures. Across all materials, the analytical focus was not on clinical outcomes or individual health experiences, but on how intermediaries are situated within organizational environments through which migrant workers negotiate information, services, and problem-solving pathways.

### 5.2 Analytical approach

A thematic approach was appropriate because the study sought to identify recurrent patterns in how intermediary roles and institutional arrangements were described across heterogeneous qualitative sources, rather than to reconstruct individual biographies or generate a formal grounded theory. We audio-recorded and transcribed formal interviews and group discussions in the language of data collection. The first author documented field-based conversations and expert consultations in research notes and coded them within the same thematic framework. Later in the analysis, the first author translated selected Mandarin-language quotations into English, and the second author reviewed the translations for clarity and consistency of meaning. The first author also checked summary interpretations with all participants to clarify ambiguities and confirm that their accounts were represented accurately.

Analytically, formal interview and group discussion transcripts constituted the primary corpus. Field-based conversations and expert consultation notes were also coded within the same thematic framework and used to assess, contextualize, and refine emerging interpretations. Because these materials were generated through different forms of engagement and were not equivalent in depth or format to the formal interviews, they were treated as supplementary rather than interchangeable sources. Policy and administrative documents, by contrast, were not coded but were used to clarify institutional arrangements and contextualize participant accounts.

Initial coding involved close readings of transcripts to identify recurrent expressions, interactional patterns, and contextually grounded descriptions of health-related navigation. These inductive codes were then refined into broader thematic groupings that captured intermediaries' institutional positioning and the mechanisms through which they shape migrants’ capacity to access, communicate, and manage health needs. NVivo 14 was used to organize transcripts and retrieve coded segments; however, theme development relied on interpretive analysis rather than software-driven procedures.

The progression from codes to themes did not follow a predefined theoretical template. After inductive theme development, the SEM was incorporated heuristically to structure interpretation. In this post hoc analytic stage, SEM provided a scaffold for situating emergent themes across individual, interpersonal, organizational, community, and policy levels. Its use was not tied to data collection or initial coding but clarified how institutional arrangements intersect with migrant health navigation across multiple contexts.

Interpretive rigor was supported through cross-perspective contextualization across contributor types, including academic scholars with institutional expertise, NGO practitioners with frontline experience, and field-based perspectives from migrant community settings. This cross-perspective engagement enabled examination of themes from multiple vantage points without implying formal comparison between cases. The first author conducted the initial coding and developed preliminary thematic groupings. Both authors then discussed emerging themes and interpretations, revisiting relevant transcript segments when interpretations needed clarification or refinement. This iterative process ensured the final themes remained grounded in the empirical material.

The first author conducted all interviews and field engagement and thus had direct contact with participants and the settings represented in the data. Trained in health policy, her approach to the study was informed primarily, but not exclusively, by the SEM, and she led the initial coding and empirical theme development. The second author did not participate in data collection and contributed primarily to thematic refinement and conceptual interpretation, drawing on the sociology of health. We considered these differences in disciplinary orientation and proximity to the field during joint analytic review; when interpretations differed or appeared to extend beyond participants’ accounts, we returned to the transcript or research-note material before refining and agreeing on the themes.

### 5.3 Ethical considerations

This study was approved by the Social and Behavioral Research Ethics Committee at National Taiwan University (Protocol No. 202311HS034). All procedures complied with national regulations and recognized ethical principles for social and behavioral research, including voluntary participation, informed consent, confidentiality, and secure data handling. We conducted formal interviews and group discussions under the approved study protocol. Later field-based conversations and expert consultations arose during the research process and remained within the ethical scope of the approved project; we obtained informed consent before each conversation regarding the study purpose, the intended use of their contribution, privacy protections, and their right to decline questions or withdraw, and these additions did not require a formal protocol amendment.

All participants received written information outlining the study purpose, their role, and measures to protect their safety and well-being. No identifying information was collected unless explicitly permitted. All transcripts and research notes were anonymized and stored on password-protected drives with access restricted to members of the research team. No financial incentives were provided. Interviews were conducted either face-to-face or via secure videoconferencing platforms, at the participants’ preference, with particular attention to privacy and risk minimization.

## 6 Findings

This section presents findings from two analytically parallel case studies: Taiwan and Japan. Each case is organized using the SEM and examines intermediary roles across five levels: individual, interpersonal, organizational, community, and policy. The two cases are presented as separate institutional contexts rather than direct comparisons. Interview and group discussion accounts form the core of the findings, while field-based and consultative contributions are drawn selectively where they clarify particular institutional or community-level dynamics. The analysis also notes recurring mechanisms to clarify how intermediary governance arrangements shape migrant health navigation in each setting.

### 6.1 Taiwan case study

#### 6.1.1 Individual level

At the individual level, Taiwanese participants described constraints affecting migrant workers’ health navigation, including information asymmetry, language barriers, and financial pressure, particularly during early stages of employment. T-3 consistently described intermediaries as a key “bridge between workers and employers,” particularly when workers navigate labor regulations, healthcare systems, and job-related communication while lacking “knowledge of local laws, cultural norms, and labor rights.” This positioning makes intermediaries a key channel through which workers interpret health-related information and options.

At the same time, intermediaries’ control over information can restrict workers’ ability to verify employment conditions or resolve problems independently. T-4 noted that intermediaries may “restrict direct communication between workers and employers,” contributing to discrepancies between recruitment promises and actual job duties, such as being recruited for eldercare but assigned childcare responsibilities. Taiwanese participants indicated that limited direct access to employers could make it difficult for workers to clarify expectations or negotiate changes.

Language mediation further illustrates this dual role. While agencies’ translation assistance facilitates initial navigation, both T-3 and T-4 described how relying on it may increase dependence on agencies when formal language training and alternative support are limited. Financial arrangements further compounded these constraints. T-4 also described recruitment-related fees and debt as limiting workers’ capacity to change jobs or exit unfavorable conditions.

The field-based conversation with migrant participant T-6 provided a more direct illustration of how such constraints may enter care-seeking decisions. T-6 described workers sometimes enduring physical discomfort rather than seeking medical attention because of time constraints, out-of-pocket costs, or dismissive responses that framed symptoms as ordinary tiredness rather than requiring care. As a single field-based account, this observation is not treated as representative; however, it illustrates how financial and relational constraints can converge at the point of health decision-making.

These accounts illustrate the ambivalence of informational mediation—intermediary assistance can reduce linguistic and procedural barriers while simultaneously concentrating interpretive authority in the same actors on whom workers depend. Health-related access is therefore shaped not only by whether information is available, but by who controls its interpretation and transmission.

#### 6.1.2 Interpersonal level

At the interpersonal level, intermediaries mediate relationships between migrant workers and employers, shaping communication, dispute resolution, and access to assistance. Taiwanese participants described employers as often depending on intermediaries to manage administrative procedures and workplace communication because of bureaucratic complexity and language barriers. As T-3 observed, “most employers just ask the intermediary to handle everything,” a reliance that can shift responsibility away from employers and consolidate intermediary authority.

This delegation extended beyond employment administration. Participants described employers relying on intermediaries to arrange medical visits, address insurance-related procedures, and communicate health concerns. T-5, for example, interpreted this pattern as a displacement of responsibilities that might otherwise fall to employers. In this sense, intermediation did not simply facilitate access; it redistributed responsibility for workers’ health-related needs, while potentially distancing employers from direct accountability.

Intermediaries often interpret workplace instructions, mediate conflicts, and determine how concerns are conveyed. T-3 and migrant participant T-6 described how workers’ access to assistance may depend on their relationship with the intermediary; those viewed favorably were more likely to receive help, including support in arranging medical visits. Conversely, T-4 emphasized that “when conflicts arise, the outcome often depends on whose side the intermediary takes,” highlighting the asymmetrical power embedded in these relationships.

Control over job transfers further illustrates this dynamic. T-4 emphasized that the transfer document (轉出函) required for changing employers is typically managed by intermediaries. T-4 also described workers as often lacking the knowledge or capacity to initiate transfers independently, allowing intermediaries to retain substantial influence over mobility and, by extension, access to alternative working and living conditions relevant to health.

These interpersonal dynamics show how intermediaries operate as power brokers, shaping everyday interactions in ways that condition migrant workers’ autonomy and capacity to navigate health-related needs.

#### 6.1.3 Organizational level

At the organizational level, intermediaries in Taiwan operate within a transnational recruitment system that links formal agencies with informal brokers across sending and receiving countries. Taiwanese participants described how Taiwanese agencies collaborate with *niu tou* (牛頭; informal or local recruiters/brokers) in origin countries to recruit workers through personal networks. These arrangements, according to T-3, may generate financial obligations and intermediary dependence before migration begins.

T-4 noted that many *niu tou* are former migrant workers familiar with policy loopholes, which can facilitate recruitment practices that may limit workers’ bargaining power. Because recruitment and placement processes span multiple jurisdictions, both T-4 and consulted scholar T-5 described oversight as complicated and fragmented, allowing intermediary practices to operate with limited accountability.

The accounts of both T-3 and T-4 indicated that financial arrangements could reinforce intermediary influence over recruitment and placement. Financial relationships between agencies and employers could shape hiring and placement decisions, while workers with fewer financial resources could face more constrained options or reduced support. These organizational dynamics position intermediaries as powerful institutional actors shaping recruitment flows, financial arrangements, and labor accessibility—conditions that indirectly structure migrant workers’ exposure to risk and their capacity to navigate health-related challenges.

#### 6.1.4 Community level

At the community level, public attitudes and regulatory responses influence the environment in which intermediaries operate, often reinforcing their role. In our conversations, T-3 and T-4 suggested that framing migrant workers as populations requiring heightened control can increase regulatory complexity and, in turn, reliance on intermediaries to manage compliance. As T-3 observed, during the COVID-19 pandemic, this reliance intensified as intermediaries assumed responsibility for enforcing restrictions and coordinating employer compliance in the absence of sufficient state capacity.

Alongside these dynamics, migrant workers have developed informal community networks that provide emotional support, health information, and peer guidance. Community informant T-2 described how workers share information about hospitals, medication, and employer communication through dormitory and workplace networks. Some participants also noted attempts to organize collectively, including union-related activities, as strategies to reduce dependence on intermediaries.

These accounts suggest that intermediarization can also be institutionally produced by regulation itself—as compliance requirements multiply, intermediary actors become increasingly necessary as interpreters and implementers of administrative demands. Such community-level dynamics reflect a dual landscape: institutional arrangements that amplify intermediary authority, and migrant-led networks that offer alternative pathways for information and support.

#### 6.1.5 Policy level

At the policy level, Taiwanese participants articulated divergent views on the role and regulation of intermediaries within Taiwan’s migrant labor system. Some emphasized transparency and worker empowerment, arguing that clearer information and administrative knowledge would enable workers to make informed choices and reduce reliance on exploitative agencies (e.g., T-3). Others advocated stronger state-led alternatives, contending that intermediaries restrict autonomy by controlling job options and information flows (e.g., T-4).

As proposed by the consulted scholar T-1, a more pragmatic position acknowledged intermediaries’ deep institutional embedding and emphasized incremental reforms through clearer service guidelines and stricter monitoring. Despite differing views on whether intermediaries should be regulated, competed out, or structurally replaced, these perspectives converged on a more fundamental point: dependence on intermediaries is sustained by administrative complexity and the limited availability of viable alternative support infrastructures. There was also broad agreement that existing reforms have not substantially reduced structural reliance on intermediaries, leaving recruitment and placement processes largely intermediary-driven.

Taken together, the Taiwan case illustrates how intermediary arrangements are embedded across multiple contexts of migrant workers’ social ecology, shaping the institutional conditions under which health-related navigation becomes possible, constrained, or deferred.

### 6.2 Japan case study

#### 6.2.1 Individual level

At the individual level, Japanese participants described constraints affecting migrant workers in Japan related to language adaptation, healthcare access, and employment mobility, with these constraints shaped by the distinct institutional designs of the TITP and the SSW system. Under the TITP, workers typically arrive with limited language preparation, as pre-departure training is “very basic, sometimes only a few days,” leaving many unable to understand workplace instructions or navigate everyday systems, according to J-4. While SSW applicants must pass language and skills examinations before entry, participants in one group discussion emphasized that post-arrival support is often minimal, particularly in smaller workplaces.

Language barriers directly affect health navigation. Some migrant workers may avoid seeking medical care because they “don’t know how to explain their symptoms” and lack access to interpretation support, as J-2 noted. Even when formal entitlements to care exist, communication difficulties and unfamiliarity with procedures can delay treatment or lead to care avoidance.

Health-related vulnerabilities were described as particularly acute among TITP workers. At one group discussion, J-1 and J-3 mentioned that concerns about missing work, financial costs, or employer reactions could lead some workers to delay seeking care even when experiencing serious symptoms. J-3 further described women workers as facing additional risks, including limited support in cases of sexual harassment or pregnancy, with fears of dismissal or repatriation discouraging reporting.

Employment mobility further conditions individual-level agency. In one group discussion, participants emphasized that transitioning from TITP to SSW is difficult because of mismatches in skill standards and certification timelines, while access to SSW Type 2, which permits long-term residence and family reunification, remains highly restricted. As J-4 noted, “the bar is so high that only a few workers ever reach it,” potentially constraining long-term planning among workers seeking to remain in Japan. These findings show how institutional designs shape individual capacities to interpret options, manage risk, and seek assistance, positioning intermediaries and support structures as central to whether health needs are addressed or deferred.

#### 6.2.2 Interpersonal level

At the interpersonal level, intermediary arrangements have structured communication and support differently across the TITP and SSW systems. Under the TITP, Supervising Organizations function as formal intermediaries, managing interactions between workers and employers and serving as the primary channel for problem reporting. While this mediation can reduce misunderstandings, J-4 noted that communication is often filtered, making it difficult for workers to raise concerns directly.

By contrast, participants perceived support under the SSW system as less consistently accessible through intermediary organizations than under the TITP. Although SSW entails different formal support arrangements, they emphasized that workers may, in practice, bear greater responsibility for negotiation and problem-solving when such support is difficult to access, particularly because of language barriers and workplace power asymmetries. As J-4 explained, “if SSW workers run into problems, they’re on their own.”

Healthcare navigation illustrates this trade-off. TITP workers may receive logistical assistance from Supervising Organizations in arranging medical visits or insurance procedures, easing access but reinforcing dependence. In one group discussion, participants noted that they perceived comparable support as less accessible under the SSW system and described greater uncertainty around healthcare procedures and communication, which could discourage care-seeking.

These dynamics reveal a tension between mediated support and greater responsibility for self-navigation: structured intermediation can facilitate navigation while constraining voice, whereas greater responsibility for direct interaction may enhance independence but increase isolation and risk.

#### 6.2.3 Organizational level

At the organizational level, Japan’s migrant labor governance reflects contrasting assumptions about oversight and responsibility. The TITP operates through a supervisory infrastructure involving multiple actors, including sending-country organizations and Japanese Supervising Organizations. According to J-4, although these bodies monitor compliance and worker welfare, workers may indirectly bear some associated costs, while the concentration of supervisory functions can obscure accountability.

The SSW system does not reproduce the same supervisory structure as the TITP and organizes employment and support through different institutional arrangements. J-4 considered that practical support was less consistently mediated in some settings, with workers and employers assuming greater responsibility for navigating disputes, administrative procedures, and health-related needs where organized support was limited.

J-2 and J-3 described coordination between Japanese agencies and sending-country recruiters as shaping worker selection and placement in ways that could limit workers’ influence over job allocation and conditions. At the time of data collection in early 2024, the Japanese government had already adopted a policy to replace the TITP with a new Employment-for-Skill-Development (ESD) Program, including expanded opportunities for workers to change employers (Immigration Services Agency of Japan, 2024a). Legislation establishing the ESD Program was subsequently promulgated in June 2024 (Immigration Services Agency of Japan, 2024b). Japanese participants expressed uncertainty about whether these reforms would meaningfully alter existing power relations.

Overall, organizational arrangements in Japan shape the degree and form of intermediary involvement, with direct consequences for how migrant workers manage employment and health-related challenges.

#### 6.2.4 Community level

At the community level, participants emphasized that limited integration infrastructure contributes to social isolation among migrant workers, particularly those in restrictive visa categories. As J-2 stated, short visa durations, limited family reunification options, and workplace-centered living arrangements constrain opportunities for community participation and informal support.

In one group discussion, participants noted that without robust community-based services, migrant workers rely heavily on informal peer networks to exchange information about healthcare, employment rights, and coping strategies. However, these networks remain uneven and insufficient to compensate for gaps in institutional support, particularly for workers facing acute health or employment crises. These community-level conditions reinforce reliance on intermediaries or employers as primary points of contact, narrowing alternative pathways for information and assistance.

#### 6.2.5 Policy level

At the policy level, persistent gaps have existed between formal entitlements and practical access, as our participants highlighted. Under the TITP, Supervising Organizations are assigned substantial responsibilities for worker support. However, J-3, for instance, expressed concerns about these organizations’ independence, especially where close coordination with sending-country recruiters creates conflicts of interest. Other Japanese participants described the SSW system as placing greater emphasis on worker autonomy, while institutional assistance for navigating administrative or health-related procedures was perceived as less consistent.

Healthcare policy illustrates this disjuncture. Accounts from participants, especially J-2 and J-3, indicated that, despite formal eligibility for health insurance, complex documentation requirements and limited multilingual assistance could hinder migrants’ effective use of these services. As a result, formal inclusion does not consistently translate into functional access.

Participants also highlighted the lack of standardized evaluation mechanisms to assess the effectiveness of migrant labor programs. J-2 mentioned that without systematic monitoring, implementation gaps persist, and reform efforts risk remaining symbolic rather than transformative. Therefore, the Japan case shows how policy designs redistribute responsibility for health navigation without necessarily ensuring capacity, producing varying configurations of intermediary involvement and institutional support that shape migrant workers’ everyday experiences.

## 7 Discussion

While prior applications of the SEM framework have emphasized destination-country determinants such as linguistic competence, healthcare accessibility, and community resources (Moyce et al., 2023; Richard et al., 2011; Rivera, 2023), the findings of this study indicate that migrant health navigation is shaped by an institutional landscape that SEM has rarely foregrounded: the intermediary environment through which procedural, administrative, and informational operations are routed. In both Taiwan and Japan, intermediaries do not provide medical treatment, yet they routinely influence what information is accessible, when care is feasible, and how bureaucratic uncertainty is resolved. These findings suggest that intermediaries do not function merely as interpersonal actors; collectively, they also shape an organizational environment that conditions migrants’ practical capacity to seek care.

The study identifies two distinct institutional configurations through which *intermediarization* occurs. In Taiwan, intermediaries hold de facto responsibility for procedural and administrative support across recruitment, employment, and everyday coordination. In Japan, comparable functions are embedded within formal institutional designs under the TITP and the SSW system. Despite these differences, the health-related implications converge. In both contexts, intermediary-mediated pathways affect how migrant workers interpret information, manage documentation, and navigate access to healthcare.

Healthcare access, therefore, reflects not only healthcare system design but also the organizational architectures migrants attempt to use to meet everyday needs. This interpretation is consistent with direct narrative research among overseas Filipino workers in Taiwan’s electronics and technology sector, where communication difficulties, legal and financial constraints, and broker-related barriers shaped decisions to seek care, forgo care, or leave health concerns unaddressed (Antonio and Lee, 2024). These patterns resonate with qualitative research on migrant workers in Japan, showing how institutional hierarchies, linguistic demands, and visa precarity generate chronic stress and constrain health-related decision-making even without explicit barriers to care (Asis and Carandang, 2020).

Notably, several constraints identified in this study—including recruitment-linked debt, reliance on bureaucratic intermediaries, and institutional ambiguity—originate upstream in transnational recruitment environments structured by intermediary arrangements. Bilateral labor agreements (BLAs) further show how transnational governance can shape these environments. Kurlander and Cohen (2022) conceptualize BLAs as operating through meso-level institutionalization processes involving migration networks, intermediaries, and sector-specific arrangements, showing that similar regulatory instruments may develop differently across employment sectors. Although the present study did not systematically analyze BLAs, such arrangements matter because they allocate responsibilities for recruitment, monitoring, support, and accountability across states, employers, and intermediary actors, shaping how health-related needs are addressed or left unattended.

Such dynamics are complex to capture when SEM is applied primarily as a model of host-country contextual determinants. This insight aligns with migration infrastructure scholarship, which conceptualizes brokerage not as a discrete economic function but as a distributed institutional constellation spanning states, markets, and social actors (McKeown, 2012; Xiang and Lindquist, 2014). The Taiwan and Japan cases thus do more than illustrate SEM dynamics; they expose an intermediary environment in which state functions, employer expectations, and migrant needs intersect to shape health navigation.

By foregrounding this intermediary environment, the study suggests that SEM-based analyses of migrant health can be strengthened by selectively engaging with migration infrastructure perspectives. Such integration does not redefine SEM but extends its interpretive reach by accounting for how health inequities are produced not only through healthcare systems or community resources, but also through upstream recruitment structures and intermediary-mediated administrative processes that span borders. The findings indicate that intermediary arrangements constitute an organizationally embedded and transnationally configured institutional environment that shapes migrants’ practical ability to seek care yet remains under-described in conventional SEM applications.

Recognizing this intermediary environment allows us to conceptualize the dynamics observed here as *health intermediarization*: an institutional process in which access to health-related information, assistance, and care is organized through intermediary arrangements. Across the two settings, this operates through several recurrent mechanisms—informational mediation, through the filtering and interpretation of knowledge; procedural mediation, through documentation and administrative navigation; relational mediation, through the organization of communication among workers, employers, and institutions; and the redistribution of responsibility for resolving health-related problems across migrants, employers, intermediaries, and public actors.

Therefore, health intermediarization does not simply refer to the presence or absence of brokers; it describes how the practical conditions of health navigation are institutionally mediated, including where intermediary support is uneven, delegated, or partially withdrawn. Framed in this way, the study does not propose a new model but renders analytically visible an institutional process that has remained backgrounded within existing frameworks. This clarification offers a critical understanding of migrant health inequities and provides a basis for reflecting on the implications of intermediary governance for both health research and migration policy.

## 8 Conclusion

The findings indicate that migrant workers’ access to healthcare is shaped not only by formal entitlements within national health systems but also by the organizational environments that mediate bureaucratic tasks, information interpretation, and procedural navigation. Whether delegated to private brokers in Taiwan or configured through the distinct institutional arrangements of Japan’s TITP and SSW systems, these intermediation structures condition the practical feasibility of seeking care in ways that remain analytically backgrounded when SEM applications focus primarily on destination-country determinants.

Recognizing the role of this intermediary environment in health navigation clarifies that barriers to care arise not only at the point of clinical interaction but also upstream within recruitment-linked administrative and procedural processes. The conceptual position developed here—interpreting these dynamics as *health intermediarization*—does not propose a new theoretical model but extends SEM’s interpretive reach by making this institutional process explicit. By selectively integrating insights from migration infrastructure scholarship, the analysis shows how transnational governance of labor mobility interacts with local organizational conditions, with implications for migrant health equity.

Several limitations should be noted. First, the study primarily draws on the observations and perspectives of scholars, NGO practitioners, and other institutional or community-based contributors and was not designed to systematically examine migrant workers’ lived experiences. Thus, future research should examine health intermediarization more directly from migrant workers’ perspectives and explore how intermediary-mediated experiences vary across different institutional settings.

Second, the composition and modes of data collection differed between the Taiwan and Japan cases, and the study did not include intermediary actors themselves, employers or government officials. Sectoral coverage was also uneven: while the Taiwan material included examples from industrial work, domestic and care work, and fisheries, the Japan material centered predominantly on formally employed workers in service and industrial sectors and did not substantively capture the circumstances of fishery or live-in care workers. These asymmetries limit the range of perspectives represented, and the different modes of data collection may also have shaped interactional dynamics and the degree of contextual detail available, particularly because the Japanese sessions were conducted online whereas the Taiwan material included in-person field engagement; this further supports treating the cases as analytically parallel rather than directly comparable. Hence, the analysis cannot establish whether the same forms of health intermediarization operate in equivalent ways across occupational settings.

Finally, the analysis focuses on migrant workers within formally regulated labor-migration pathways. The forms and consequences of health intermediarization may vary for undocumented migrants and across skill categories or other migration arrangements in which regulatory structures and reliance on intermediaries differ. These boundaries define the scope of the present analysis and warrant further investigation across various migrant populations and labor-migration contexts.

Overall, advancing migrant health equity requires attention not only to healthcare systems themselves, but to the intermediary-mediated environments through which migrants manage paperwork, access information, and negotiate uncertainty. Making these environments analytically visible strengthens our understanding of how health inequities are produced and sustained; it underscores the importance of examining organizational and governance contexts alongside clinical access in migrant health research.

## Ethics approval statement

Ethical approval for this study was granted by the Social and Behavioral Research Ethics Committee at National Taiwan University (Protocol No. 202311HS034). All participants provided written informed consent.

## Funding statement

The study was self-funded. Publication-related costs are partially supported by the Federal Ministry of Education and Research (BMBF) and the Baden-Württemberg Ministry of Science under the Excellence Strategy of the German Federal and State Governments, as well as by the Population Health and Wellbeing Research Center from the Featured Areas Research Center Program within the Higher Education Sprout Project of the Ministry of Education (MOE) in Taiwan (No. NTU-115L900401).

## AI declaration in the manuscript preparation process

During the preparation of this work, we used Grammarly and ChatGPT for proofreading academic English writing. No new content was produced with the use of generative AI technology. We reviewed and edited the draft as needed and take full responsibility for the published content.

## Ethics declaration

Written informed consent to participate in the study and for the use of anonymised contributions in publication was obtained from all participants. The privacy rights of participants have been observed.

This study was performed in compliance with relevant laws, regulatory frameworks and guidelines where the research took place. This study was approved by the National Taiwan University Social and Behavioral Research Ethics Committee (Approval No. 202311HS034).

## CRediT authorship contribution statement

**Yen-Ru Chen:** Writing – review & editing, Writing – original draft, Project administration, Methodology, Investigation, Formal analysis, Data curation, Conceptualization. **Po-Han Lee:** Writing – review & editing, Validation, Supervision, Resources, Methodology, Funding acquisition, Formal analysis, Data curation, Conceptualization.

## Declaration of competing interest

The authors reported no potential conflict of interest.

## Data Availability

Deidentified coded data derived from the interview materials may be made available upon reasonable request. Audio recordings have been destroyed, and full transcripts cannot be shared as participant consent for transcript dissemination was not obtained.
